# Molecular testing for gastrointestinal pathogens in intestinal tissue of infants with necrotizing enterocolitis or spontaneous intestinal perforation

**DOI:** 10.1038/s41372-024-01999-z

**Published:** 2024-06-07

**Authors:** Maria M. Talavera-Barber, Pablo J. Sánchez, Miriam Conces, Irina Kaptsan, Kathy Everhart, Amy Leber, Daniel T. Malleske, Mohannad Moallem, Santiago Panesso-Gómez, Masako Shimamura

**Affiliations:** 1 Department of Pediatrics, Avera Research Institute and University of South Dakota Sanford School of Medicine, Sioux Falls, SD USA; 2 Department of Pediatrics, Division of Neonatology, Nationwide Children’s Hospital and The Ohio State University College of Medicine, Columbus, OH USA; 3 Center for Perinatal Research, Ohio Perinatal Research Network, Abigail Wexner Research Institute at Nationwide Children’s Hospital, Columbus, OH USA; 4 Department of Pediatrics, Division of Pediatric Infectious Diseases, Nationwide Children’s Hospital and The Ohio State University College of Medicine, Columbus, OH USA; 5 Department of Pathology, Nationwide Children’s Hospital and The Ohio State University College of Medicine, Columbus, OH USA; 6 Center for Vaccines and Immunity, Abigail Wexner Research Institute at Nationwide Children’s Hospital, Columbus, OH USA; 7 Department of Pathology and Laboratory Medicine, Nationwide Children’s Hospital, Columbus, OH USA; 8 Department of Pediatrics, Division of Neonatology, Children’s Hospital of Philadelphia, Philadelphia, PA USA; 9 Department of Pediatrics, Division of Neonatal-Perinatal Medicine, Indiana University School of Medicine, Indianapolis, IN USA; 10 Department of Gynecology Oncology, Magee-Womens Research Institute, University of Pittsburgh, Pittsburgh, PA USA

**Keywords:** Gastrointestinal diseases, Disease model, Acute inflammation

## Abstract

**Objective:**

The objective of this study was to determine the frequency of common gastrointestinal bacterial, parasitic, and viral pathogen detection in necrotizing enterocolitis (NEC) or spontaneous intestinal perforation (SIP) -associated intestinal tissue.

**Study design:**

Retrospective cohort study examined formalin fixed, paraffin embedded (FFPE) surgical or autopsy intestinal tissue from NEC or SIP specimens. DNA and RNA were extracted and analyzed by multiplex PCR panel (GIFA Biofire). DNA or RNA from stool samples containing each pathogen were extracted for positive controls.

**Results:**

The total number of intestinal tissue samples were 193 from 310 infants (156 NEC, 37 SIP). Six (3%) infants with stage III NEC tested positive for a target pathogen; 2, *C. difficile;* 3, *Enteroaggregtive E. coli*; and 1, *Giardia*. No gastrointestinal viral pathogens were detected.

**Conclusion:**

Molecular testing yielded few GI pathogens suggesting that these organisms are likely not major causes or facilitators of NEC or SIP.

## Introduction

Necrotizing enterocolitis (NEC) and spontaneous intestinal perforation (SIP) are major causes of gastrointestinal morbidity and mortality in preterm infants [1, 2]. While NEC is associated with intestinal dysbiosis [3, 4], formula feeding [5], intestinal ischemia [6], and prematurity [7], SIP is a distinctly different entity that primarily affects extremely premature infants during the first week of age [2]. The histopathological hallmark of NEC is mucosal necrosis, whereas SIP is characterized by mucosal hyperplasia with an absence of the muscularis layer that leads to intestinal perforation [8]. The mechanisms of injury in both conditions remain poorly understood, but underlying inflammation has been implicated in their pathogenesis.

Microorganisms colonizing the preterm gastrointestinal tract may contribute to the pathogenesis of NEC and SIP [9]. Studies of infant stool have revealed dysbiotic signatures of the bacterial microbiome before the onset of NEC, while preclinical models of NEC have shown the absence of disease in germ-free animals [10, 11]. Pneumatosis intestinalis, the radiographic and pathologic hallmark of NEC, is believed to occur secondary to gas formation from Gram-negative bacteria, specifically *Clostridioides spp*., within the intestinal wall [12]. In addition, NEC cases in neonatal intensive care units (NICUs) can occur in clusters [13] or may be associated with seasonal viral outbreaks [14, 15].

A number of published case reports and case series describe gastroenteritis-causing pathogens that were detected during NEC in individuals or NICU populations. Organisms such as *Salmonella* [16], *Clostridioides species* [17], *E.coli spp*. [18], *Rotavirus* [19], *Campylobacter* [20], *Shigella* [20], and *Adenovirus* [21] have been reported in association with NEC in preterm infants (sTable 1). Although some of these reports were longitudinal or case-control studies, the significance of the pathogens reported in small case series is not well understood. Pathogens were identified using stool culture, immunoassays, or polymerase chain reaction (PCR), with some studies using 16S ribosomal RNA or metagenomic sequencing to define microbial communities in infants with and without NEC. Molecular PCR-based diagnostic panels have been developed to facilitate the detection of intestinal pathogens causing community-acquired gastroenteritis in children and adults, but these tools have not been applied to determine the prevalence of such organisms among preterm infants with NEC or SIP. The goal of the study was to analyze a large archive of pathology specimens from cases of NEC or SIP cases in preterm infants using a PCR-based panel to define the frequency of molecular detection of gastroenteritis-causing intestinal pathogens which were previously identified in the literature in association with NEC [22, 23].

## Methods

### Study population

**Fig. 1:**
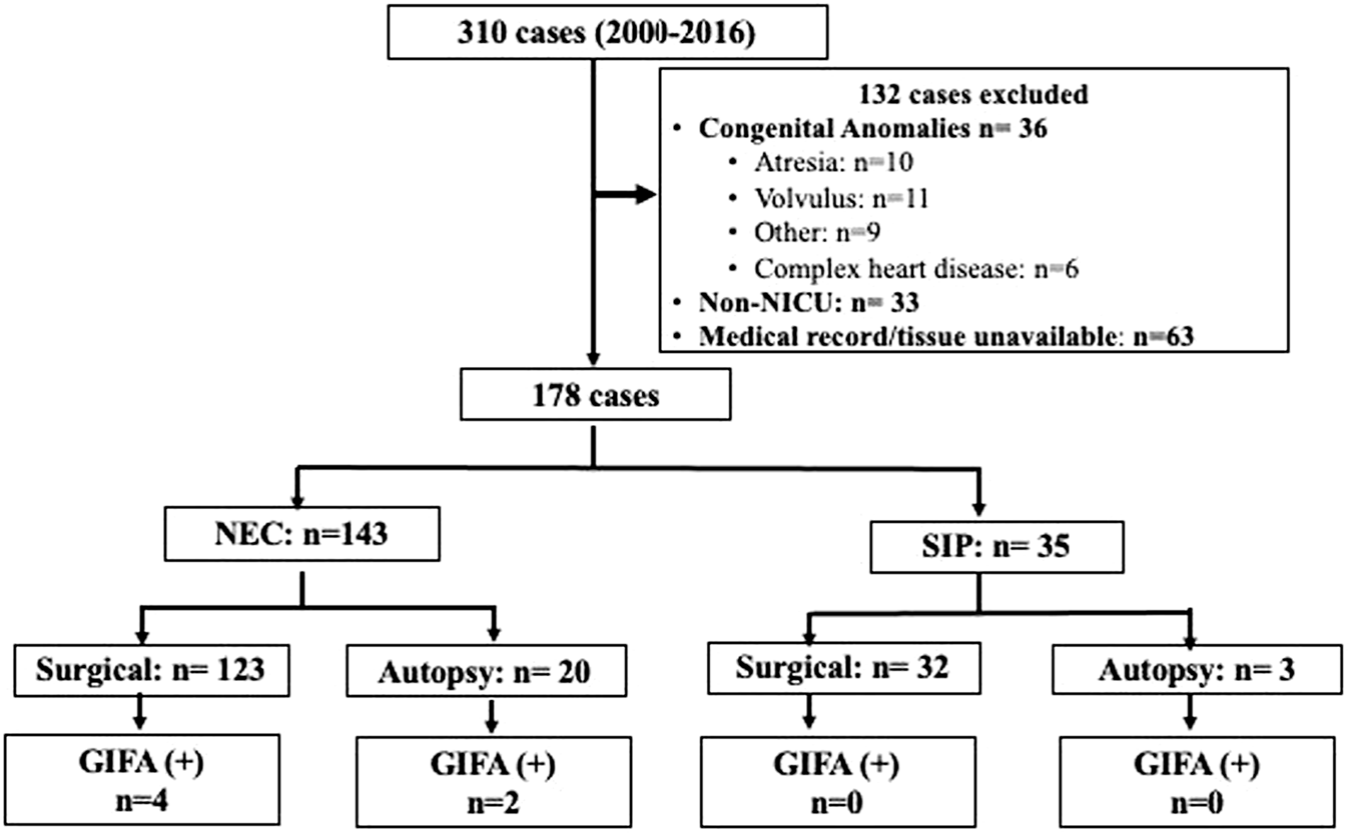
Schematic description of study population. The study population included 178 (57%) of infants with histopathologically confirmed NEC (*n* = 143) and SIP (*n* = 35). Cases of NEC or SIP were classified by outcome (surgical or autopsy). “Other” exclusions consisted of ileal strictures, omphalocele, and abdominal hernia. NICU neonatal intensive care unit, NEC necrotizing enterocolitis, SIP spontaneous intestinal perforation, GIFA Gastrointestinal FilmArray.

This was a retrospective cohort study of infants with surgical NEC (Bell’s ≥ 2B) [24] or SIP who were treated in the Level 4 NICU at Nationwide Children’s Hospital (NCH), Columbus, OH from 2000 to 2016. This study was approved by the NCH Institutional Review Board (IRB) (IRB15-00553). The study leveraged existing specimens from a previously published study examining the detection of cytomegalovirus (CMV) in intestinal tissue of preterm infants with NEC or SIP [25]. As in our prior study, cases were identified by review of the surgical pathology database of the Pathology Department, using the diagnoses of “necrotizing enterocolitis” or “small bowel perforation.” NEC or SIP was confirmed after histopathologic review by a pediatric pathologist. Inclusion criteria were: (i) histopathological-confirmed diagnosis of NEC or SIP; and (ii) sufficient paraffin-embedded tissue available in the pathology archive. Exclusion criteria were: (i) histopathologic diagnosis of non-NEC or SIP gastrointestinal disease (e.g. atresia, volvulus, omphalocele, gastroschisis); (ii) presence of congenital heart disease; (iii) no retrievable specimens in the pathology archives; or (iv) non-NICU patients (Fig. 1).

The medical records of infants were reviewed for pertinent demographic, clinical, laboratory, and radiographic data, and clinical outcomes including retinopathy of prematurity, bronchopulmonary dysplasia (BPD), and short bowel syndrome.

### Tissue processing and FilmArray^®^ gastrointestinal panel (GIFA) (Biofire^®^ Diagnostics, Salt Lake City, UT) testing

Table 1 lists the gastrointestinal pathogens included in the GIFA panel [22]. Paraffin-embedded intestinal tissues were processed and DNA and RNA were extracted as previously described [25]. For each patient sample, 10 μl of DNA and 20 μl of RNA were combined with 700 μl of sample buffer and analyzed off-label using the multiplex GIFA PCR panel (BioFire® Diagnostics, St. Lake City Utah).

**Table 1:** GIFA organism profile.

Bacteria	Diarrheagenic *Escherichia Coli/Shigella*	Viruses	Parasites
*Campylobacter (jejuni, coli, and upsaliensis*	*Enteroaggregative E.coli (EAEC)*	Adenovirus F40/41	*Cryptosporidium*
*Clostridium difficile (toxin* *A/B)*	*Enteropathogenic E.coli (EPEC)*	Astrovirus	*Cyclospora cayetanensis*
*Plesiomonas shigelloides*	*Enterotoxigenic* *E.coli (ETEC) It/st*	Norovirus GI/GII	*Entamoeba histolytica*
*Salmonella*	*Shiga-like toxin producing E.coli* *(STEC) stx1/stx2 E.coli O157*	Rotavirus A	*Giardia lamblia*
*Yersinia enterocolitica*	*Shigella/Enteroinva sive E. coli (EIEC)*	Sapovirus (I, II, IV, and V)	

*Vibrio (parahaemolyticus, vulnificus, and cholerae)*

*Vibrio cholerae*

De-identified stool samples with positive clinical testing for GI pathogens were acquired from the clinical microbiology laboratory at NCH for use as positive assay controls, including the following pathogens: *Campylobacter spp*., *Clostridioides difficile*, *Salmonella, Yersinia enterocolitica, Enteroaggregative E. coli (EAEC), Shiga-like toxin producing E.coli (STEC), Shigella/Enteroinvasive E. coli (EIEC), Cryptosporidium*, *Giardia lamblia*, norovirus, sapovirus, adenovirus, astrovirus, and rotavirus. Stool samples without clinically identified GI pathogens were used as negative controls. Nucleic acids were extracted from stool samples in identical fashion as for paraffin-embedded tissues, except for omission of the paraffin lysis step. For the extracted control nucleic acids, 10 μl of DNA and 20 μl of RNA were mixed with 700 μl of sample buffer and tested using the GIFA panel to validate pathogen detection using this nucleic acid extraction method. All stool analyses were performed at a research laboratory at NCH.

### Statistical analyses

Descriptive analyses were used to summarize patients’ demographic characteristics using means with standard deviation or medians (interquartile ranges) and frequency distributions as appropriate. Categorical variables were analyzed using Chi-square or Fisher’s exact tests, and continuous variables using *T*-test or Mann–Whitney tests according to data distribution. Correlations were performed using Spearman’s rank correlation coefficient since most of the data did not follow a normal distribution. All analyses were performed using Prism 9.0 (GraphPad Software, LaJolla CA). Two-sided *p*-values < 0.05 were considered statistically significant.

## Results

From 2000 to 2016, of 310 infants identified in the Pathology database with a diagnosis of “necrotizing enterocolitis” or “small bowel perforation,” 178 (57%) cases had either NEC (*n* = 143; 80%) or SIP (*n* = 35; 20%) based upon intraoperative surgical reports, histopathologic confirmation of the diagnosis by pathologist review, and availability of formalin-fixed, paraffin-embedded tissue (Fig. 1). Of the 143 infants with NEC, intestinal tissue samples had been obtained at the time of surgery for NEC (86%, *n* = 123) or at autopsy (14%, *n* = 20). Among the 35 infants with SIP, 32 (91%) intestinal tissue samples had been obtained at surgery while 3 (9%) were from autopsy. The GIFA detected pathogens in 6 (4%) of the 143 NEC cases including 4 from surgical and 2 from autopsy specimens (Fig. 1). The detected GI pathogens included *Clostridioides difficile* (*C. difficile*, *n* = 2), *Enteroaggregative E.coli* (EAEC, *n* = 3), and *Giardia lamblia* (*G. lamblia*, *n* = 1). No viral gastrointestinal pathogens included in the GIFA were detected in NEC or SIP cases. No pathogens were detected by GIFA in intestinal tissue of SIP cases. Assay validation after stool nucleic acid extraction was confirmed with positive GIFA tests for clinically identified pathogens in all positive control stool specimens and negative tests for all negative control stool specimens.

For infants with NEC, infectious evaluations typically included blood, urine, cerebral spinal fluid (CSF) cultures, and in certain cases peritoneal fluid cultures. For GIFA (+) NEC cases, only 2/6 or 33% had a positive blood culture result. The urine, CSF and peritoneal fluid cultures were negative for all 6 patients. GIFA (−) NEC cases had 33/137 or 23% positive blood cultures, 2% positive urine cultures, 1.5% positive CSF cultures, and 5.9% positive peritoneal cultures. GIFA (–) SIP cases had 8/35 or 23% positive blood cultures and negative urine, CSF, and peritoneal cultures (Table 2). Positive blood cultures for the GIFA (+) cases included *coagulase negative Staphylococcus* (*CONS*) and *E.coli*, the latter of which corresponded with a *EAEC* GIFA+ test in the same infant (Table 3), suggesting that the *EAEC* detected by GIFA was a NEC-related pathogen rather than a contaminant or incidental finding. For the GIFA (−) NEC cases, pathogens in the blood cultures were not included as microbes tested in the GIFA panel except for the *E.coli spp*. detected in one blood culture but not the GIFA.

**Table 2:** Clinical Infectious Evaluation for GIFA-positive and GIFA-negative patients.

Culture site	GIFA (+) NEC	GIFA (−) NEC	GIFA (−) SIP
	*N* = 6	*N* = 137	*N* = 35
Blood	2 (33%)^a^	33 (24%)^b^	8 (23%)^f^
Urine	0	8 (5.9%)^c^	0
CSF	0	3 (2.2%)^d^	0
Peritoneal Fluid	0	2 (1.5%)^e^	0

^a^*Coagulase-negative Staphylococcus (CONS), Escherichia coli (E.coli)*

^b^*E.coli, Methicillin-sensitive Staphylococcus aureus (MSSA), Staphylococcus epidermidis (S. epidermidis), Pseudomonas aureginosa (P. aureginosa), Candida albicans (C. albicans), Klebsiella pneumoniae (K.pneumoniae), CONS, Serratia. marcescens (S. marcescens), Clostridioides butyricum (C. butyricum), Enterobacter aerogenes (E.aerogenes), Enterobacter cloacae (E. cloacae), Enterococcus faecalis (E. faecalis), Methicillin-resistant Staphylococcus aureus (MRSA)*

^c^*P. aureginosa, Clostridioides difficile (C. difficile), Clostridioides perfingens (C. perfingens), E. cloacae, Candida glabrata (C.glabrata), E.coli, K. pneumoniae, Bacteroides fragilis (B. fragilis)*

^d^*K. pneumoniae, E.coli, Klebsiella oxytoca (K.oxytoca)*

^e^*MSSA, K.pneumoniae*

^f^*MSSA, C. albicans, CONS, C. glabrata, S. epidermidis, E. cloacae*

**Table 3:** Maternal and Infant Characteristics of GIFA-positive NEC cases.

Cases	GIFA Pathogen	Blood cultures	GA (weeks)	BW (g)	DOL at diagnosis	PCA (weeks)	NEC Stage/Tissue	Feeding	Sequelae d/t NEC	Maternal parity	Mode of delivery	Chorioamnionitis
1	*C. difficile*	No growth	31	1755	10	33	IIIb/Colon^a^	formula	none	G2P2	Vaginal	N
2	*C. difficile*	unknown	32	1513	10	34	IIIb/Cecum^a^	EBM	SBS	G2P2	C/section	N
3	*EAEC*	No growth	31	1204	21	34	IIIb/Cecum^a^	formula	death	G3P3	C/section	N
4	*EAEC*	*CONS*	25	715	23	28	IIIb/Cecum^a^	formula	none	G3P3	Vaginal	Y
5	*EAEC*	*E.coli*	34	2597	5	34	IIIb/SI^b^	EBM	death	G5P5	Vaginal	N
6	*G. lamblia*	No growth	30	1145	9	31	IIIb/SI^b^	EBM	death	G2P2	C/section	N

*CONS* *coagulase-negative Staphylococcus*, *C. difficile* *clostridioides difficile*, *EAEC* *enteroaggregative E.coli*, *E.coli* *Escherichia coli*, *G. lamblia Giardia lamblia*, *GA* gestational age, *BW* birth weight, *DOL* day of life, *PCA* post-conceptual age, *SI* small intestine, *EBM* expressed breastmilk, *SBS* short bowel syndrome, *BPD* bronchopulmonary dysplasia, *C/section* Caesarean section

^a^Surgical specimen

^b^Autopsy specimen

### Characteristics of GIFA-positive NEC cases

Gestational ages of GIFA-positive patients ranged from 25 to 34 weeks and birth weight ranged from 715 grams to 2597 grams, and the age of onset of NEC ranging from 5 days to 23 days postnatal age or by post conceptual age of 28–34 weeks which aligns with peak presentation of 32 weeks as shown by Yee et al. [26] (Table 3). There was no difference in breastmilk vs. formula feeding among the GIFA-positive cases. One case (*EAEC)* was delivered to a mother with chorioamnionitis.

### Clinical and laboratory characteristics of GIFA-positive NEC patients

We compared the clinical and laboratory characteristics of GIFA-positive NEC cases (*n* = 6) and GIFA-negative NEC (*n* = 137) and GIFA-negative SIP (*n* = 35) patients (Table 4). There were no differences among the GIFA-positive and GIFA-negative groups in demographic characteristics of gender, race or ethnicity, nor were there differences in gestational age or birth weight (Table 4). There was no difference in any human milk feedings between the GIFA-positive and -negative groups. We then investigated clinical signs and symptoms at the time of NEC or SIP presentation that included (i) acute abdominal changes including distention, tenderness or absent bowel sound, (ii) bloody stools, (iii) stability on room air or need for respiratory support, and (iv) gastric residuals. There were no differences in presenting symptomatology between both groups. Analysis of specific laboratory findings (complete blood count and liver function tests) revealed no differences in complete blood count at the time of NEC or SIP presentation among each group. However, there was significantly higher (*p* = 0.027) direct bilirubin in infants with GIFA-negative NEC + GIFA-negative SIP compared to those with GIFA-positive testing (Table 4). Major sequelae from NEC diagnosis in GIFA-positive cases included death (*n* = 3, 50%), bronchopulmonary dysplasia (BPD, *n* = 1) and short bowel syndrome (SBS, *n* = 1) (Table 4).

**Table 4:** Clinical and laboratory characteristics of GIFA-positive and GIFA-negative NEC/SIP patients.

Demographics	GIFA-positive NEC	GIFA-negative NEC	GIFA-negative SIP
	(*n* = 6)	(*n* = 137)	(*n* = 35)
Age at NEC or SIP diagnosis, days (IQR)	10 (8–22)	9 (5–25)	8 (7–24)
Male gender (%)	4 (67)	78 (57)	20 (54)
Race/ethnicity (%)			
White	3 (50)	85 (62)	21 (59)
Black	2 (33)	32 (23)	10 (30)
Hispanic	1 (17)	8 (6)	2 (5)
Biracial	0 (0)	5 (4)	1 (3)
Asian	0 (0)	1 (1)	0 (0)
Other	0 (0)	5 (4)	1 (3)
Gestational age, weeks (IQR)	31 (29–33)	27 (23–32)	29 (24–31)
Birth weight, grams (IQR)	1359 (1038–1966)	1035 (700–1811)	1122 (904–1219)
Human milk diet (any) (%)	4 (67)	74 (54)	18 (51)
Mortality (%)	2 (33)	52 (38)	12 (33)
Clinical Signs at the time of NEC/SIP (%):			
Abdominal distention	5 (83)	126 (92)	31 (89)
Gastric residuals	3 (50)	42 (31)	14 (40)
Bloody stools	2 (33)	55 (40)	16 (46)
Respiratory support			
Room air	2 (33)	25 (18)	12 (34)
Mechanical ventilation	3 (50)	101(74)	22 (63)
CPAP	1 (17)	11 (8)	1 (3)
Absent bowel sounds	3 (50)	96 (70)	22 (62)
Abdominal tenderness	5 (83)	126 (92)	33 (94)
Complete Blood Count, Worst Value:			
White blood cells (#/mm^3^) (IQR)	7400	21,000	12,000
	(1475–32,200)	(7600–34,900)	(4200–16,100)
Neutrophil (IQR)	25 (7–59.3)	43 (21–64)	29 (12–42)
Band (IQR)	16 (8.8–25.8)	12 (5–20)	7 (3–12)
Lymphocytes (IQR)	33.5 (10–57.3)	24 (18–31)	21 (16–28)
Metamyelocytes (IQR)	2.5 (0.8–6)	1 (0–3)	2 (0–4)
Platelets (# x 1000/mm^3^) (IQR)	92.5 (50.3–271)	80 (42–180)	110 (60–136)
Liver Function, (Worst Value):			
ALT (U/L) (IQR)	21 (16.3–24.3)	40.2 (24.6–91.3)	33.6 (27–33.1)
AST (U/L) (IQR)	34.5 (31.3–38.5)	56.2 (38.1–133.6)	52 (28.2–76.5)
Total bilirubin (mg/dL) (IQR)	3.3 (1.2–10.3)	5.7 (2.9–9.3)	3.6 (2.1–4.5)
Direct bilirubin (mg/dL) (IQR)^*^	0.4 (0–0.8)	2.6 (0.9–5.1)	1.3 (0.4–1.8)
Complete Blood Count (At NEC Diagnosis):			
White blood cells/mm^3^ (IQR)	8500	9600	7500
	(2280–19,530)	(5600–17,600)	(3450–11,610)
Neutrophil (IQR)	13.5 (4.3–40)	31 (8–46)	16 (3–22)
Band (IQR)	22.5 (12–38.5)	18 (4–22)	20 (11–28)
Lymphocytes (IQR)	31 (25–61.8)	35 (22–48)	28 (18–31)
Metamyelocytes (IQR)	7 (0–9)	6 (1–9)	2 (0–5)
Platelets (# x 1000/mm^3^) (IQR)	175 (136–234)	106 (67–215)	161 (90–280)
Retinopathy of Prematurity	0/3 (0)	25/71 (34)	5/18 (34)
BPD	1 (16)	22 (16)	4 (12)
Short Bowel Syndrome (SBS)	1 (17)	31 (23)	3 (9)

*GIFA* Gastrointestinal Film Array, *NEC* necrotizing enterocolitis, *SIP* spontaneous intestinal perforation, *SGA* small gestational age, *CPAP* continuous positive airway pressure, *ALT* alanine aminotransferase, *AST* aspartate aminotransferase, *BPD* bronchopulmonary dysplasia

*p = <0.05; Mann–Whitney rank sum test

### Perinatal characteristics of GIFA-positive cases

All mothers of the GIFA-positive patients were multiparous with ≥ 2 gravid status (Table 3). There was no significant difference among the different types of pathogens detected and the mode of birth delivery (50% vaginal delivery, 50% caesarean section).

## Discussion

The contribution of intestinal pathogens causing community-acquired gastroenteritis to the pathogenesis of NEC or SIP remains unknown. In this study, using a commercially available multiplex PCR-based assay, we detected *C. difficile*, *Enteraggregative E.coli* (*EAEC*), and *G. lamblia* in 6 of 143 infants with NEC Stage ≥2B but no pathogens in 35 SIP cases. Blood culture results from one GIFA (+) case corresponded with an *E.coli spp* likely indicating that *EAEC* was a NEC-related pathogen and not a contaminant; possibly via intestinal translocation during the acute phase of the disease. Although we previously detected CMV by PCR or immunohistochemistry (IHC) in the same intestinal tissue samples biorepository [25], CMV is not included in the GIFA panel and no viral gastrointestinal pathogens were detected using GIFA. A study by Ullrich et al. similarly investigated the presence of gastrointestinal pathogens in 22 NEC ileal samples compared to 15 non-NEC controls using a different multiplex-PCR panel [27]. This study investigated the same common gastrointestinal pathogens as our study, however, results did not show the presence of any viral, bacterial or protozoan pathogens in NEC intestinal tissue [27]. Our larger subject cohort and, perhaps, differences in test sensitivity of the GIFA could have contributed to the difference in our results. Our findings indicate that typical GI pathogens are unlikely to be primary causes of NEC, but may support a rationale for further investigation into the role of gastrointestinal pathogens in inflammation during NEC. Our results also indicate that microbes are unlikely to contribute to SIP pathogenesis.

Case studies describing the detection of infectious community acquired pathogens in stool samples of preterm infants with NEC date back to the 1970’s and 1980’s with *Salmonella*, *Enterotoxigenic E.coli*, *Clostridioides difficile* and *rotavirus* being the most commonly detected pathogens in cases (sTable 1). Although several studies were conducted using prospective or case-control methods, many are case reports that have a risk for reporting bias and lack a comparator group to establish the prevalence of the reported pathogen as agents associated with NEC in general. Prior studies used culture, immunoassay and PCR-based techniques to isolate bacterial pathogens from stool samples to describe the possible association and/or colonization of these pathogens with the onset of NEC. Only one prior study used a multiplex PCR assay to detect GI pathogens, and that study only analyzed 28 tissues [27]. To better define the association of these reported pathogens with NEC or SIP, we employed a commercially available microarray platform (GIFA) to investigate the presence of community acquired gastrointestinal pathogens in intestinal tissue affected by NEC or SIP to determine the association of these pathogens with the development of these diseases in a large cohort of 193 preterm infants.

Studies have shown that the NICU environment (length of hospitalization) and antibiotic exposures were major influencing exposures in preterm neonates [28]. Several *Clostridioides* species, *C. neonatale* [29]*, C. difficil* [17], and *C. perfringens* [30], isolated from blood, stool, and peritoneal samples have been associated with NEC outbreaks. One longitudinal study described the preterm gut microbiome in those who developed NEC compared to controls as temporally distinguished by the abundance of *Clostridioides* compared to *E.coli*. Stool samples from infants with early onset NEC (defined as <14 days of age) were characterized by an abundance of *Clostridioides species* (mainly *C. sensu stricto)* vs. those who developed late-onset NEC with an increased abundance of Gammaproteobacteria (*E.coli* and *Shigella*) [31].

The association of *E.coli* subtype colonization and NEC has been described in several studies. A case report described of the presence of *E.coli* O157:H7 in a term infant who developed NEC [32]. Preclinical animal models of NEC have demonstrated opposing effects of *E.coli subtypes* on disease severity. Thomas et al describes how the colonization of the commensal strain *E. coli* EC25 protected against experimental NEC [33], while Roy et al demonstrated in a higher level preclinical model in piglets that *E.coli*-fermented short chain fatty acid metabolites in formula induced a more severe form of NEC that mimicked the human form of the disease [34]. Other studies have characterized the preterm gut microbiota as less diverse in those that develop NEC [35] with a potential influence of uropathogenic *E. coli* as a risk factor for increased severity of NEC and death [36]. To date, there are no reported cases of *G. lamblia* associated NEC cases in the literature. However, *Giardia* infection has been shown to alter the human bacterial microbiome structure and function inducing a dysbiotic environment after the enteropathogenic organism has been cleared [37]. Although our study did not detect common viral gastrointestinal pathogens (e.g. rotavirus, norovirus, astrovirus), a recent meta-analysis did find a significant association between CMV, rotavirus, norovirus, and astrovirus infection and increased risk for NEC [38].

Limitations of our study include a single center study with retrospectively collected samples from subjects requiring surgical intervention or with fatal outcome. We also note that the GIFA panel is indicated for stool samples and limits the pathogen detection to those included in the panel. Its use on formalin-fixed tissue is not included in the FDA label and has undetermined performance characteristics. Furthermore, the processing of formalin-fixed tissue may have impacted the sensitivity of pathogen detection [39]. To address these limitations, we validated the nucleic acid extraction process for formalin-fixed, paraffin-embedded tissue by utilizing the identical extraction protocol for known positive stool specimens and demonstrated positive detection of target pathogens by GIFA.

In summary, we acknowledge that this is a largely negative study with the detection of a few pathogens (*C. difficile, E.coli subtype-EAEC* and *G. lamblia)* in intestinal tissues from cases of severe NEC, making them unlikely primary causes of NEC. However, the presence of these pathogens may provide insight into the role of common gastrointestinal pathogens as possible infectious facilitators of intestinal inflammation leading to NEC in some preterm infants, and may deserve further study in animal models or NICU populations.

## Supplementary information

Supplementary table 1

## Data Availability

The data that supports the findings in this study are available from the last author (MS), upon request.
